# Longitudinal Changes of Motor Function in Becker Muscular Dystrophy

**DOI:** 10.1212/NXG.0000000000200285

**Published:** 2025-07-28

**Authors:** Luca Bello, Pietro Riguzzi, Giuliana Capece, Martina Penzo, Angela Petrosino, Elena Sogus, Sara Mastellaro, Michela Caroli, Matteo Villa, Daniele Sabbatini, Domenico Gorgoglione, Sara Vianello, Gianni Sorarù, Elena Pegoraro

**Affiliations:** 1 Department of Neuroscience DNS, University of Padova, Italy; and; 2 Department of Cardiac, Thoracic, and Vascular Science and Public Health DSCTV, University of Padova, Italy.

## Abstract

**Background and Objectives:**

Becker muscular dystrophy (BMD) is due to *Duchenne muscular dystrophy* gene variants allowing partial expression of dystrophin. A detailed description of disease trajectories in different genetic subgroups, and the identification of factors predicting progressive vs stable disease, are indispensable for designing and interpreting current and future clinical trials.

**Methods:**

We recruited male participants with a molecularly confirmed diagnosis of BMD at our Institution, and followed them up with an observational longitudinal design with functional evaluations, including North Star Ambulatory Assessment (NSAA), 6-minute walk test, and timed function tests.

**Results:**

We recruited 107 participants. Time-to-event analyses of age at loss of ambulation estimated that only 25% of individuals with BMD lose ambulation by age 60 years. Functional measures, over a follow-up of a mean ± SD of 6.4 ± 3.5 evaluations per participant, and a time of 6.1 ± 3.6 years, showed a poor performance in the common deletions del 45–47 and del 45–48, and preserved muscle function with del 48 and deletions ending on exon 51. In the overall cohort, all measures declined significantly over time, but this decrease was more evident in genetic groups with more marked weakness, and in participants with baseline values of NSAA of 32/34 or lower.

**Discussion:**

These data refine genotype-phenotype correlations in BMD; quantify the decline in several practical and reliable motor outcome measures, which can be directly applied to power calculations for clinical trials; and point to useful inclusion/exclusion criteria for trials. Long-term outcomes will serve as a comparator for “real-world” efficacy data of upcoming therapeutics.

## Introduction

Becker muscular dystrophy (BMD), Online Mendelian Inheritance in Man (OMIM) #300376, is a neuromuscular disorder caused by variants in the *Duchenne muscular dystrophy* (*DMD*) gene, which allow the expression of abnormal and/or quantitatively defective dystrophin protein in skeletal muscle fibers.^1^ Such variants are most commonly in-frame deletions of 1 or more *DMD* exons, but also include in-frame duplications, small insertion/deletions, missense, intronic, and nonsense variants.^2,3^

The clinical spectrum of BMD spans different phenotypes,^4^ including “subclinical” cases who are completely asymptomatic or presenting only with elevated serum creatinkinase (CK), postexertional myalgia, or occasional rhabdomyolysis (“pseudometabolic” phenotype); “benign” cases with mild proximal weakness; “moderate” cases with waddling gait, difficulty climbing stairs, and getting up from the floor; and “severe” cases with loss of independent ambulation. Dilated cardiomyopathy (DCM) is a major complication of BMD^5^ and may arise not only in individuals with clinically evident muscle weakness but also in those with subclinical or benign muscle phenotypes.^6^ Respiratory insufficiency, on the other hand, is rare in BMD, and mostly observed in advanced and severe cases.^7^ Finally, cognitive and neuropsychiatric issues such as language delay, intellectual disability, and autism spectrum disorders may affect a portion of the BMD population due to altered expression of CNS dystrophin isoforms.^8^

As more and more potential treatments emerge for dystrophinopathies,^9-13^ the identification of efficient outcome measures, as well as the description of disease trajectories and prognostic factors, become paramount for designing clinical trials for BMD. In 2016, we described longitudinal changes over 1 year of some DMD-derived outcome measures, such as the North Star Ambulatory Assessment (NSAA), the 6-minute walk test (6MWT), and timed function tests (TFTs)^14^; since then, other groups have described BMD cohorts followed up to 2 or 3 years,^15,16^ confirming a measurable progression of muscle weakness and motor function impairment, especially in individuals with moderate to severe phenotypes, e.g., as observed with the most common *DMD* in-frame deletions, including exons 45–47 and 45–48.

Here, we expand both sample size and follow-up duration of our previously reported cohort,^14^ aiming to describe long-term disease trajectories, refine genotype-phenotype correlations also for the less common genetic groups, and accurately estimate the rate of progression of motor impairment in BMD subgroups with specific genetic and clinical features.

## Methods

### Ethics Statement

All evaluations involving participants and experiments involving muscle tissue samples were performed in accordance with relevant guidelines and regulations and were approved by the Padova Ethics Committee for Clinical Experimentation (protocols 4032/2016 and 5310/2022 at Azienda Ospedale Università Padova). All participants or their legal guardians provided written informed consent to study procedures.

### Inclusion Criteria

Participants were selected from the database at the European Reference Network Center for Neuromuscular Diseases at Azienda Ospedale-Università Padova. Inclusion criteria were as follows:

1. Male participants with a molecularly confirmed diagnosis of BMD, defined as an in-frame *DMD* variant, or present but quantitively or qualitatively altered dystrophin protein, as assayed by immunoblot (IB) or immunofluorescence of muscle biopsy tissue (excluding revertant fibers and trace amounts, i.e., less than 3% of control), with any pathogenetic *DMD* variant;
2. Availability of clinical data (longitudinal functional measures, and/or age at loss of ambulation (LoA), sometimes obtained retrospectively).

Inclusion criteria did not include clinical features, such as age at LoA. Therefore, individuals with severe phenotypes, classically corresponding to “intermediate dystrophinopathy”, i.e., LoA between the age of 13 and 16 years, and even DMD, i.e., LoA before 13 years, were not excluded a priori. Similarly, there were no exclusion criteria related to corticosteroid or immunosuppressive treatments.

The cohort described here represents an expansion in sample size, as well as a prolongation in follow-up time, of that described in our previous report of 1-year longitudinal changes.^14^

The molecular diagnosis of BMD was performed with one of the following techniques: multiplex Polymerase Chain Reaction (PCR) with defined deletion boundaries, Multiplex Ligation-dependent Probe Amplification (MLPA), or sequencing (Sanger or next generation sequencing, NGS). Single exon deletions were confirmed with 2 independent methods. Dystrophin immunoblot (IB) was performed as described,^14^ using DYS2 as a primary antibody.

LoA was defined as age at continuous wheelchair use and evaluated in the retrospective cohort of 107 participants.

Motor function was evaluated by trained physiotherapists with the NSAA,^17^ 6MWT,^18^ and TFTs: time to rise from the floor (TRISE), timed to walk/run 10 m (TTRW10), and time to climb 4 standard steps (TSTEPS), as described,^14^ in a clinical research setting, between October 2011 and June 2023. The difference in distance between the first and last 3 minutes in the 6MWT was used as an indicator of fatigue during the test. Timed test results are expressed as velocities, to assign a “zero” value to participants unable to perform the task, and to indicate the decline in function with negative values. Participants with “zero” values because of loss of function were excluded from subsequent longitudinal analyses. Participants who had active symptoms of DCM (e.g., exertional dyspnea or dizziness, symptomatic arrhythmias, lower limb edema) were excluded from functional evaluations.

### Genetic Grouping

In our previous study,^14^ we had divided participants into 4 genetic groups: “del 45-x”, comprising deletions starting from exon 45; “del x-51”, grouping deletions ending in exon 51; “del 48”, corresponding to deletion of exon 48; “other” consisting in all other participants. Based on the larger sample size in the present study, we grouped deletions starting at exon 45 (45–47, 45–48, 45–49, and 45–55) separately. The deletion of exons 45–51 was included in the “del x-51” group, which was maintained as such, because all these deletions consistently presented a very mild skeletal muscle phonotype. We also analyzed separately the deletions of exons 48–49 and nonsense variants. Therefore, we were able to describe longitudinal functional data in 7 most relevant genetic groups: del 45–48, del 45–47, del 48, del x-51, del 45–55, del 48–49, and nonsense.

### Statistical Analyses

Distributions of quantitative and ordinal variables were described as mean ± SD, or median and range, as appropriate. Time-to-event variables were described with the Kaplan-Meier method. Quantitative/ordinal variables were compared between groups with the Kruskal-Wallis test. Linear mixed models were used to estimate the yearly decrease of functional measures in the overall population and in subgroups, with calculation of *p* values based on the Satterthwaite method. Linear mixed model estimation of yearly changes was limited to data points pertaining to adults (older than 18 years) because functional measures may be stabilized or even improve during the developmental age. Correlations between quantitative/ordinal variables were tested with the Pearson or Spearman methods, as appropriate. R v. 4.0.5 was used for statistical analyses and graphical representation. Statistical significance was set at *p* < 0.05, with no formal correction for multiple testing, given the descriptive nature of the study design and the strong correlations between the observed outcomes.

### Data Availability

Anonymized data not published within this article will be made available by request from any qualified investigator.

## Results

**Figure 1:**
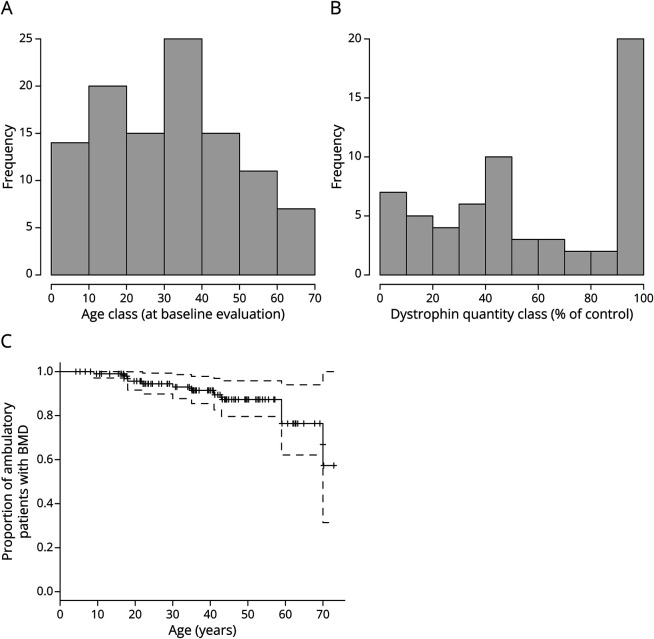
Overall BMD Cohort at Baseline (A) Histogram of the age distribution of the overall BMD cohort at baseline. (B) Histogram of the distribution by classes of dystrophin quantity, assessed by immunoblot in participants with available muscle tissue. (C) Kaplan-Meier plot of age at loss of ambulation; crosses indicate censored participants, i.e., those who were still ambulatory at the time of last evaluation; while dashed lines represent 95% CIs. BMD = Becker muscular dystrophy.

Participants who met our inclusion criteria were 107, of whom 104 had longitudinal functional data available (longitudinal cohort) and 3 only retrospective data (retrospective cohort). Average age was 31.4 years at baseline (Table 1). Distribution by age class in shown in Figure 1A, panel A.

**Table 1:** Participant Demographics and Dystrophin Quantity by Genotype Group

Variant groups			Age (y) at baseline			Dystrophin IB (% of control)		
	Specific variants		n	Mean ± SD	Median (range min ∼ max)	n with available immunoblot	Mean ± SD, (%)	Median (range min ∼ max), (%)
5' (proximal) hotspot deletions (13.1%)	N-terminal domain	del 2-7	3	37.5 ± 21.2	27.0 (23.6–62.0)	1	40 ± NA	NA
		del 3-4	1	NR	NA	0	NA	NA
		del 3-7	3	23.2 ± 7.7	27.6 (14.2–27.6)	1	10 ± NA	NA
		del 3-9	2	NR	NR	1	100 ± NA	NA
	Proximal rod domain	del 13	1	NR	NA	1	35 ± NA	NA
		del 10-25	2	NR	NR	2	100 ± 0%	100 (100–100)
		del 10-29	1	NR	NA	1	100 ± NA	NA
		del 11-30	1	NR	NA	1	100 ± NA	NA
3' (distal) hotspot deletions (69.2%)	del 45-46		1	NR	NA	0	NA	NA
	del 45-47		17	31.7 ± 16.0	34.6 (3.5–55.9)	6	45 ± 31	50 (10–90)
	del 45-48		17	39.2 ± 15.8	38.3 (9.2–69.7)	12	66 ± 26	55 (40–100)
	del 45-49		2	NR	NR	1	10 ± NA	NA
	del x-51 (45–51, 48–51, 50–51, 34–51)		15	24.7 ± 15.0	21.1 (4.5–50.7)	9	83 ± 29	100 (20–100)
	del 48		12	26.8 ± 22.1	14.5 (6.1–67.8)	8	72 ± 32	80 (20–100)
	del 45-55		5	40.6 ± 26.1	51.6 (6.9–67.3)	3	83 ± 15	80 (70–100)
	del 48-49		4	38.6 ± 25.4	45.1 (4.0–60.3)	3	57 ± 31	50 (30–90)
	del 48-57		1	NR	NA	1	30 ± NA	NA
Duplications (4.7%)	dup 16, 56–78, 19–41, 13–42, 35-42		5	26.5 ± 16.5	34.6 (5.9–44.8)	4	45 ± 21	45 (19–70)
Nonsense (3.7%)	p. Lys1061*, p.Trp1281*, p.Gln1322*, p.Trp1660*		4	19.1 ± 11.4	18.1 (8.2–32.0)	3	17 ± 14	17 (3–30)
Small indels (variants with n > 1 represent sibships; 6.5%)	c.31 + 7_31+8delACinsTT		1	NR	NA	0	NA	NA
	c.676_678delAAG, p.Lys226del		3	51.7 ± 3.2	52.8 (48.1–54.1)	1	20% ± NA	NA
	c.10099_10101delGAA, p.Glu3386del		1	NR	NA	0	NA	NA
	10507_10508delAG, p.Lys3505AlaFsX8		2	NR	NR	0	NA	NA
Synonymous (sibs; 1.9%)	c.4299G >T, p.Gly1433Gly		2	NR	NR	2	20 ± 14	20 (10–30)
Missense (0.9%)	c.478A >C, p.Thr160Pro		1	NR	NA	1	10 ± NA	NA
Total			107	31.4 ± 17.3	32.0 (3.5–69.7)	62	60 ± 34	50 (3–100)

Abbreviations: del = deletion; dup = duplication; NA = not applicable; NR = not reported to avoid disclosure of identifiable information in subgroups with n < 3; IB = immunoblot; sibs = siblings.

The cohort included several familial cases, specifically 2 sets of 3 brothers, 1 pair of twins, 5 pairs of 2 brothers, 1 pair consisting of a maternal uncle and nephew, and 1 pair comprising a maternal grandfather and grandson.

In all participants, pathogenetic *DMD* variants were identified. Eighty-six (84%) had single-exon or multi-exon deletions. The most common deletions were del 45–47 (15.9%) and del 45–48 (15.9%), followed by del x-51 (14.0%), del 48 (11.2%), del 45–55 (4.7%), and del 48–49 (3.7%). Less frequent variants were grouped together, including other less common deletions (14.9%), small deletions (7.5%), duplications (4.7%), nonsense variants (3.7%), and 1 missense variant (c.478AC, p.Thr160Pro). Among small deletions, we observed an in-frame micro-deletion in 1 participant (c.10099_10101delGAA, p.Glu3386del), and 3 brothers carried the c.676_678delAAG, p.Lys226del variant. A small frameshift deletion located in exon 74 was identified in 2 brothers (c.10507_10508delAG, p.Lys3505AlaFsX8), who showed low levels of distally truncated dystrophin upon immunofluorescence. Furthermore, a synonymous variant in exon 31 (c.4299G > T, p.Gly1433Gly) was found in 2 brothers, also with reduced levels of 390 kDa dystrophin; this variant is predicted in silico to disrupt an exon splicing enhancer.^19^ All nonsense variants were located in in-frame exons in the rod domain: p.Lys1061* (exon 24), p.Trp1281* (exon 28), p.Gln1322* (exon 29), p.Trp1660* (exon 35). In all of these exons, except exon 24, nonsense variants associated with BMD phenotypes had been previously described.^3^ Frequencies and age distribution across genetic groups are presented in Table 1.

Dystrophin quantification via IB was available for 62 participants (57.9%). Mean dystrophin quantity was 60% ± 34%, with a minimum of 3% and a maximum of 100%. The distribution by dystrophin quantity (Figure 1B, panel B) showed 3 peaks: 20 participants (32%) display dystrophin levels ranging from 90% to 100% relative to controls, in 10 (16%) 50% dystrophin was observed, and 7 (11.3%) were below 10% dystrophin compared with controls. Notably, the participant with the lowest (3%) dystrophin carried a nonsense variant, while participants with 100% dystrophin belonged to the following genetic groups: proximal rod deletions (del 10–25, 10–29, and 11–30), del 48, del 45–48, del 3–9, and del x-51. Most participants with 100% dystrophin quantity had a sublicnical or benign phenotype, with the exception of 2 participants with del 45–48 and del 11–30. Detailed data are show in Table 1.

### LoA

In the retrospective cohort of 107 participants, only 12 (11.2%) had lost ambulation. Therefore, median age at LoA could not be established in the overall cohort. The Kaplan-Meier curve showed that at the age of 60 years, 25% of participants had lost ambulation (95% CI 6%–38%), while at the age of 35–41 years, only 10% of the BMD population had lost ambulation (Figure 1C, panel C).

Only some genetic groups showed LoA events: del 45–47, with 2 participants of 16 (12.5%), and a median age of 59 years; del 45–48, with 2 participants of 17 (11.8%), and a median age of 70 years; and 8 participants with rare variants: 2 small indels; 1 del 2–7; 2 del 3–7; 1 del 45–46; 1 missense (c.478 A > C, p.Thr160Pro); and 1 dup 13–42. The latter participant with duplication 13–42 had been previously described as “mildly affected”,^20^ but he later had a progressive course with LoA at 35 years of age and DCM.

### Baseline Functional Assessments

Among 107 participants comprising the cohort, 94 were tested for 6MWT, 93 for TTRW10m, 86 for TSTEPS, 78 for TRISE, and 100 for NSAA (Table 2). Participants unable to perform the test were attributed a “zero” value. For NSAA, nonambulatory participants were evaluated for items such as “lifts head from supine” and “gets to sitting”.

**Table 2:** Baseline Measures and Yearly Change Estimated by Linear Mixed Models in the Overall BMD Cohort and Main Variant Subgroups

Measure	Group	n (pts with BL eval)	BL mean ± SD	BL median (range min ∼ max)	n (>18 y with longitudinal evaluations)	n (evals)	Estimate of yearly change	SE	*p* Value
NSAA	All	100	26.5 ± 9.8	33 (0–34)	89	504	−0.64	0.04	< 0.0001
	del 45-48	15	23.1 ± 9.2	21 (3–34)	15	93	−0.78	0.07	< 0.0001
	del 45-47	16	19.8 ± 10.2	19.5 (4–33)	14	80	−0.99	0.08	< 0.0001
	del 48	15	33.8 ± 0.8	34 (31–34)	11	50	−0.03	0.01	0.0007
	del x-51	12	33.8 ± 0.4	34 (33–34)	12	63	−0.08	0.04	n.s
	del 45-55	5	29.2 ± 8.7	34 (14–34)	5	12	−0.47	0.16	n.s
	del 48-49	4	23.0 ± 7.0	21 (17–33)	3	14	−1.35	0.16	< 0.0001
	Nonsense	4	26.5 ± 10	30 (12–34)	2	24	−0.38	0.1	0.002
6MWT	All	94	427 ± 119	435 (0–721)	86	440	−4.81	0.6	< 0.0001
	del 45-48	13	407 ± 79	412 (262–526)	14	84	−7.3	0.93	< 0.0001
	del 45-47	14	348 ± 115	367 (131–575)	13	69	−10.54	1.07	< 0.0001
	del 48	15	492 ± 88	457 (386–721)	11	47	−3.04	1.47	0.05
	del x-51	12	483 ± 124	493.5 (183–656)	12	55	−2.84	1.79	n.s
	del 45-55	5	461 ± 137	452 (280–615)	5	8	3.15	1.04	n.s
	del 48-49	3	401 ± 103	407 (295–500)	3	15	−7.25	0.91	< 0.0001
	Nonsense	4	439 ± 122	423 (312–598)	3	22	−6.85	1.81	0.001
TTRW 10 m (m/second)	All	93	2.1 ± 1.2	2.0 (0.4–5.0)	84	425	−0.042	0.006	< 0.0001
	del 45-48	13	1.66 ± 0.54	1.43 (0.91–2.5)	14	79	−0.057	0.012	< 0.0001
	del 45-47	14	1.63 ± 1.04	1.11 (0.40–3.33)	13	69	−0.066	0.007	< 0.0001
	del 48	15	2.61 ± 1.19	2.50 (1.00–5.00)	11	47	−0.035	0.012	0.01
	del x-51	11	3.24 ± 1.36	3.33 (1.43–5.00)	11	49	−0.041	0.021	n.s
	del 45-55	5	2.13 ± 1.13	2.00 (1.40–3.85)	4	9	−0.058	0.012	0.01
	del 48-49	3	1.61 ± 0.81	1.43 (0.91–2.50)	3	14	−0.041	0.005	< 0.0001
	Nonsense	4	2.11 ± 0.74	2.47 (1.00–2.50)	3	22	−0.048	0.021	0.03
TRISE (s-1)	All	78	0.43 ± 0.23	0.48 (0.05–1.0)	72	317	−0.008	0.001	< 0.0001
	del 45-48	12	0.29 ± 0.15	0.30 (0.07–0.50)	13	55	−0.009	0.003	0.018
	del 45-47	10	0.34 ± 0.23	0.37 (0.05–0.77)	9	38	−0.017	0.003	< 0.0001
	del 48	14	0.58 ± 0.25	0.50 (0.30–1.00)	11	48	−0.005	0.001	0.009
	del x-51	11	0.54 ± 0.25	0.50 (0.25–1.00)	11	49	−0.011	0.004	0.019
	del 45-55	4	0.28 ± 0.10	0.33 (0.14–0.33)	3	7	−0.016	0.004	0.015
	del 48-49	3	0.28 ± 0.19	0.17 (0.17–0.5)	3	14	−0.005	0.002	0.053
	Nonsense	4	0.43 ± 0.27	0.50 (0.05–0.67)	3	15	−0.021	0.006	0.0036
TSTEPS (steps/s)	All	86	1.7 ± 1.0	1.6 (0.3–4.0)	79	382	−0.039	0.005	< 0.0001
	del 45-48	13	1.25 ± 0.64	1.00 (0.46–2.00)	14	69	−0.054	0.013	< 0.0001
	del 45-47	12	1.07 ± 0.62	1.00 (0.29–2.00)	11	54	−0.065	0.008	< 0.0001
	del 48	15	2.00 ± 0.87	1.67 (1.33–4)	11	45	−0.02	0.009	0.050
	del x-51	11	2.29 ± 1.15	2.00 (1–4)	11	54	−0.031	0.01	0.021
	del 45-55	5	1.55 ± 0.73	2.00 (0.55–2.22)	4	9	−0.038	0.024	n.s
	del 48-49	3	1.15 ± 0.31	1.33 (0.80–1.33)	3	12	−0.024	0.005	0.0014
	Nonsense	4	2.00 ± 0	2.0 (2.00–2.00)	3	20	−0.095	0.027	0.0024

Abbreviations: NSAA = North Star Ambulatory Assessment; 6MWT = six-minute walk test; TTRW10 = time to run/walk 10 m; TRISE = time to rise from the floor; TSTEPS = time to climb 4 standard steps; eval(s) = evaluation(s).

*p* Values pertain to longitudinal evaluations performed in adults (older than 18 years) and were estimated with the Satterthwaite method from linear mixed models, including all evaluations.

The average NSAA score at the initial assessment was 26.5 ± 9.8, while the median score was 33. In fact, NSAA scores were not normally distributed, but rather indicative of a “ceiling effect”. NSAA scores ranged from a minimum of 0 to a maximum of 34.

When examining genetic groups, in line with the existing literature, the del x-51 and del 48 group exhibited the highest functional performance, with an average NSAA score of 33.8 ± 0.8 and 33.8 ± 0.4, respectively. Conversely, the largest groups, i.e., del 45–48 and del 45–47, had lower NSAA scores, with an average value of 23.1 ± 9.2 and 19.8 ± 10.2, respectively.

A similar pattern, with better functional performance associated with del x-51 and del 48, as opposed to poorer and more variable performance with del 45–48 and (even more so) del 45–47, was observed for all other functional measures (details in Table 2). Participants with del 48–49 had a similar functional status to del 45–48, while those with del 45–55 performed better, although not as well as del x-51 or del 48. The Kruskal-Wallis test showed that functional differences at baseline between these genetic groups were statistically significant for all measures (NSAA: *p* < 0.0001; 6MWT: *p* = 0.008; TTRW10m: *p* = 0.002; TRISE: *p* = 0.043; TSTEPS: *p* = 0.0003).

### Longitudinal Evaluation of Motor Function

The longitudinal study involving the entire cohort consisted of an average of 6.4 ± 3.5 clinical evaluations per participant, up to a maximum of 13. The mean follow-up duration was 6.1 years (±3.6), ranging from 0 to a maximum of 11.5 years (Table 1). Participants with no longitudinal follow-up included those who were assessed only once at our Center or participants who were unable to perform the tasks under evaluation at baseline. There were a few cases of missing data because of myalgia or fatigue.

It is worth noting that some participants initiated corticosteroid treatment during the observation period, while others were already receiving the treatment at the time of their first clinical assessment. In particular, 4 participants were prescribed corticosteroids because of severe or rapidly progressing weakness (carrying deletions of exons 3–7, 11–30, 45–47, and 45–49), and 3 received prednisone as part of immunosuppressive therapy following cardiac transplantation (carrying a deletion of exons 2–7, a missense, and a nonsense variant). Here, we opted not to compare functional outcomes between corticosteroid-treated and untreated participants. This was because corticosteroid treatment is typically administered to participants with BMD with severe weakness; therefore, comparing them with untreated participants who have milder phenotypes would not provide a meaningful basis for assessing the treatment effect.

**Figure 2:**
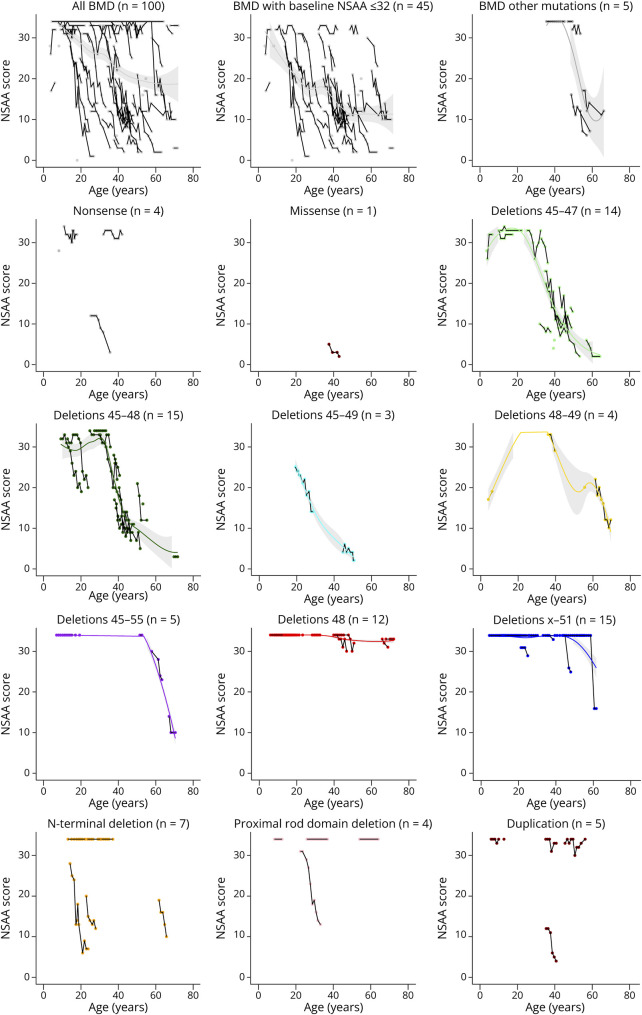
“Spaghetti” Plots of NSAA by Age, in the Overall Cohort and in Subgroups as Indicated (by Baseline Function or Genetic Group) Segments connect data points for individual participants. Tendency lines are superimposed (Lasso method); missing when data points are too variable. NSAA = North Star Ambulatory Assessment.

Longitudinal observations of NSAA over several years of follow-up allowed to capture the variably progressive nature of motor function impairment in BMD (Table 2; Figure 2). In the overall cohort, a statistically significant yearly decrease of −0.64 NSAA points was estimated, despite considerable variability and a clear ceiling effect. Such ceiling effect was mostly removed by selecting participants with baseline NSAA ≤32, i.e., with some degree of clearly measurable gross motor impairment at first evaluation. Genetic groups appeared to explain much of the variability in the progression of NSAA scores. The most numerous groups, i.e., del 45–47 and del 45–48, showed a steady decrease, estimated, respectively, at −0.99 and −0.78 points per year. In del 48, a statistically significant decrease was observed, but its magnitude of −0.03 per year may hardly be considered clinically relevant, especially if considering that most participants started from a “ceiling” value of 34. In the del x-51 group, there was no significant decrease of NSAA. In both these groups, when participants did not score a “ceiling” value of 34, the fulfillment of tasks was often hindered by exertional myalgia, or by psychiatric issues, as seen in 2 participants who had been diagnosed with a schizophrenic disorder, treated with neuroleptics, and presented with altered gait patterns or iatrogenic parkinsonism. The deletion of exons 45–55 appeared to be compatible with normal muscle strength in young men, but with some myopathic weakness later in life, as seen in 2 cases above the age of 50. Among N-terminal deletions, del 3–9 was associated with a very mild skeletal muscle phenotype in 2 unrelated participants. The group with nonsense variants exhibited a comparatively lower yearly decrease in several outcome measures in contrast to other more prevalent variants. However, given the limited number of participants within this group and their significant phenotypic variability, it is prudent to interpret this result with caution.

**Figure 3:**
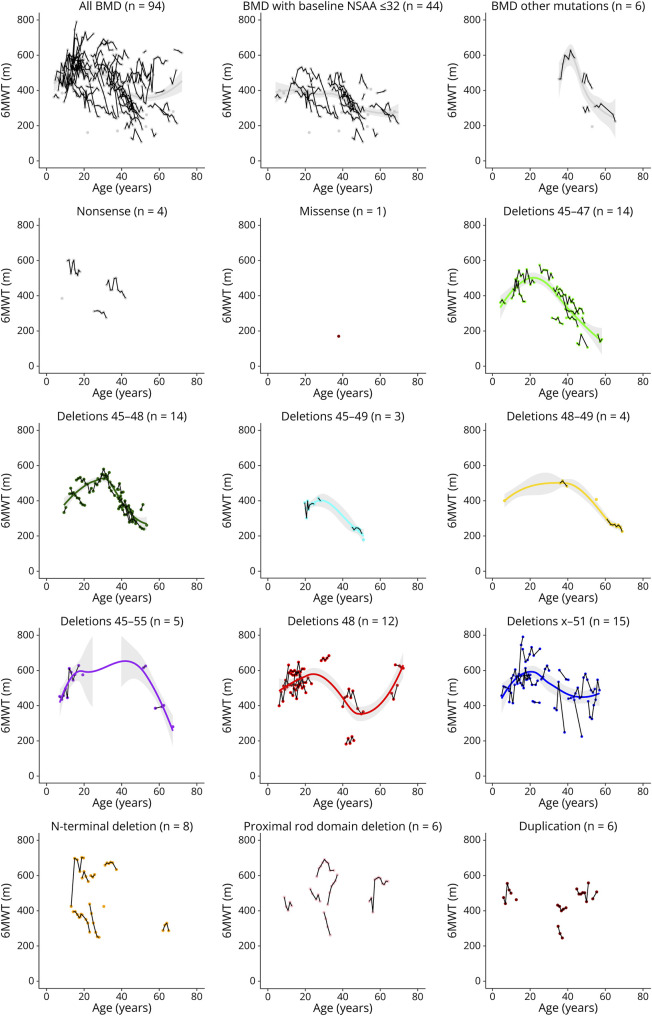
“Spaghetti” Plots of 6MWT by Age, in the Overall Cohort and in Subgroups as Indicated (by Baseline Function or Genetic Group) Segments connect data points for individual participants. Tendency lines are superimposed (Lasso method); missing when data points are too variable and do not follow a linear trend. 6MWT = six-minute walk test.

Longitudinal trajectories for the other functional measures were along the same lines as NSAA and are reported in detail in Table 2, as well as represented (for 6MWT) in Figure 3.

### Dystrophin Quantity and Functional Measures

**Figure 4:**
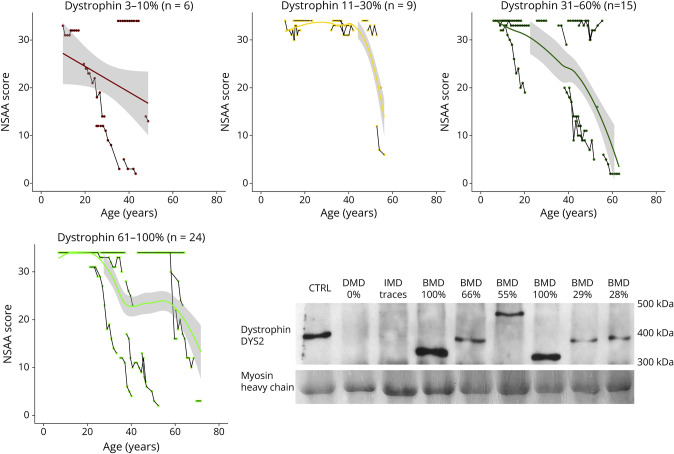
“Spaghetti” Plots of NSAA by Age, in the Overall Cohort and in Subgroups as Indicated (by Dystrophin Quantity) Segments connect data points for individual participants. Tendency lines are superimposed (Lasso method or generalized linear method as appropriate). On the lower right, an example of Western blot (WB) quantification of dystrophin is shown; samples labelled as BMD all belong to participants in this study, with the addition of a normal control, 1 DMD sample (0% dystrophin), and an IMD sample (trace amount, <3% dystrophin). BMD = Becker muscular dystrophy; DMD = *Duchenne muscular dystrophy*; IMD = intermediate (Duchenne/Becker) muscular dystrophy; NSAA = North Star Ambulatory Assessment.

Higher dystrophin quantity in participants' muscle biopsies was associated with better functional performance, as shown by a linear mixed model accounting for age, which provided significant coefficients for dystrophin quantities for most outcomes (Table 3). In these models, longitudinal functional data obtained older than 18 years were considered for all participants with available dystrophin quantity data, accounting for repeated measures in individuals as well as for increasing age. However, as it seems clear from a graphical representation of NSAA trajectories in 4 arbitrary groups of increasing dystrophin quantity (Figure 4), individual participants may present with rapidly progressive disease despite high dystrophin quantity; conversely, others, despite low dystrophin amounts, have a mild phenotype. This is probably because of the stability and functionality of different mutated dystrophin proteins, as well biological (tissue sampling) and technical variability in dystrophin quantification.

**Table 3:** Linear Mixed Model Coefficients to Evaluate the Effect of Dystrophin Quantity on Clinical Outcomes (Models Account for Age)

Measure	Estimate of change per % unit of dystrophin, adjusted by age	SD	*p* Value
NSAA score	0.055	0.04	n.s
6MWT (m)	1.45	0.45	0.002
TTRW 10 m (m/second)	0.009	0.004	0.0218
TRISE (second^−1^)	0.001	0.008	n.s
TSTEPS (steps/s)	0.007	0.003	0.0358

Abbreviations: n.s. = not significant; NSAA = North Star Ambulatory Assessment; 6MWT = six-minute walk test; TTRW10 = time to run/walk 10 m; TRISE = time to rise from the floor; TSTEPS = time to climb 4 standard steps.

*p* Values pertain to longitudinal evaluations performed in adults (older than 18 years) and were estimated with the Satterthwaite method from linear mixed models, including all evaluations.

### Fatigability

We evaluated fatigability by comparing distances walked in the first vs last 3 minutes of the 6MWT. We found that in the overall BMD cohort (n = 92, 534 evaluations), there was a decrease between the 2 halves of the test, from 226 ± 63 to 223 ± 65 m, averaging −3.0 ± 18.0 m (*p* ≤ 0.0001). This decrease was larger in 42 participants with baseline NSAA ≤32 (183 ± 48 vs 178 ± 48 m, averaging −5 ± 10 m, *p* < 0.0001, data from 258 evaluations), while there was no significant decrease in 50 participants with NSAA >32 (268 ± 46 vs 267 ± 46, averaging −1 ± 23 m, *p* = n.s., 271 evaluations). These data indicate that fatigability, expressed as a reduction in partial distances covered in the second half of the test compared with the first, is more pronounced in participants with clear muscle weakness, as assayed by NSAA.

### Predictive Value of Baseline NSAA

As shown in several analyses above, the baseline NSAA value seems to be a strong predictor of weakness progression in BMD. We analyzed NSAA decline rate with linear mixed models in different baseline NSAA classes, as detailed in Table 4. The fastest and most significant decline rates were observed with baseline NSAA between 10 and 32.

**Table 4:** Linear Mixed Model Parameters for Yearly NSAA Change in Participants Grouped by Baseline NSAA Score

Baseline NSAA score	n >18 y with longitudinal evaluations	n (evaluations)	Estimate of yearly change	SE	*p* Value
All	89	504	−0.64	0.04	<0.0001
34	36	193	−0.04	0.01	0.0043
33	8	44	−0.01	0.04	n.s
30–32	8	52	−0.95	0.15	<0.0001
25–29	4	26	−1.40	0.18	<0.0001
20–24	9	72	−1.28	0.08	<0.0001
15–19	8	49	−0.68	0.10	<0.0001
10–14	7	34	−0.96	0.12	<0.0001
5–9	4	17	−0.01	0.04	n.s
0–4	5	16	−0.27	0.12	0.048

Abbreviations: NSAA = North Star Ambulatory Assessment; SE = standard error.

*p* Values pertain to longitudinal evaluations performed in adults (older than 18 years) and were estimated with the Satterthwaite method from linear mixed models, including all evaluations.

## Discussion

Observational data regarding functional measures in BMD have been described in a few studies,^14-16^ typically in a time frame of 1–3 years. These data have informed the design of the first interventional clinical trials, i.e., those with Givinostat (NCT03238235), Vamorolone (NCT05166109), and Sevasemten (NCT05291091). As observed with DMD, demonstrating the efficacy of therapeutics in the relatively short time frame of placebo-controlled studies (1–2 years maximum) may be challenging,^21,22^ and long-term real-world data will be necessary to assess functional benefits. Therefore, long-term observational data sets with validated measures employed in clinical trials, as presented here, may be instrumental as an external comparator.

Two relevant considerations should be made about the choice of inclusion criteria for this study, which are based on genotype and dystrophin assays. First, there were no clinical criteria, which represent the most commonly used basis to distinguish BMD from DMD (i.e., LoA before or after the age of 16). Of 107 participants, only 1, carrying a hemizygous deletion of exons 2–7, lost ambulation at the age of 9, suggestive of a DMD phenotype, but with 40% dystrophin expression on IB. No participants lost ambulation between the ages of 13 and 16 years, classically corresponding to an “intermediate” phenotype. Therefore, our criteria based on genotype and dystrophin expression selected phenotypes classically corresponding to BMD in 106/107 (99.1%) cases. The only patient with a DMD phenotype did not contribute to longitudinal functional data but was included in the LoA analyses.

Second, all dystrophinopathy patients in our database with out-of-frame or truncating variants, and preserved ambulation beyond the age of 16 years, had available dystrophin quantification assays on muscle biopsy tissue; otherwise, we would have risked excluding BMD patients with truncating variants and preservation of ambulation into adult age. Specifically, males with truncating *DMD* variants (predicting DMD) and preserved ambulation beyond the age of 16 years were 20 in our Center database. Of these, 9 had >3% dystrophin expression and entered this study (2 del 3–7, 4 nonsense, 2 distal frameshift variants), while 11 had absent or trace level dystrophin, and have been included in studies of DMD genetic modifiers.

Genotype-phenotype correlations described in our previous study were confirmed and refined by data presented here. Specifically, participants carrying the most frequent deletions, particularly those involving the recombination hotspot 45–55 and starting from exon 45 (45-x), exhibited a more severe disease course, characterized by consistent progression of muscle weakness. Notably, individuals with del 45–55 represented an exception, typically showing a milder BMD phenotype. However, during the extended follow-up period, we identified disease progression in 2 participants harboring del 45–55, who presented with measurable muscle weakness past the age of 50 years.

Conversely, participants carrying deletions ending in exon 51 (del x-51), as well as those with deletion of exon 48, demonstrated better, if not normal functional performances, which remained stable throughout the extended follow-up period. In these genotype groups, only mild loss of motor function was observed in sporadic cases or older individuals, with a clinical impression that this functional impairment was primarily because of exertional myalgias or CNS involvement, which affected their ability to collaborate during clinical assessments. These individuals would be probably excluded from clinical trials because of comorbidities hindering the functional evaluations.

Less common deletions involving exons from 10 to 40 have been previously associated with mild or asymptomatic phenotypes.^4^ Consistently, participants from our cohort with deletions encompassing exons 10–25 (in 2 brothers), 11–30, and 10–29 retained ambulation throughout the entire follow-up period and demonstrated good motor performances, as indicated by NSAA score consistently exceeding 32, despite frequently reported myalgia and cramps.

By analyzing the longitudinal variation in outcome measures in relation to dystrophin levels, we observed that participants with lower dystrophin values generally achieve lower scores and experience a more rapid decline, compared to participants with higher dystrophin levels, with statistical significance in most outcome measures. However, there are frequent instances where high dystrophin levels do not correspond to preserved motor function, or vice versa.

In general, it can be stated that higher levels of dystrophin have a positive effect on muscle function, but the dystrophin quantification alone is not a strong prognostic factor in any single individual with BMD, regardless of pathogenetic variant type. In fact, understanding the relationship between dystrophin quantity and clinical phenotype is complex for several reasons. Firstly, dystrophin levels may vary significantly even among healthy controls; a variability whose physiologic bases and consequences are scarcely understood. Secondly, commonly used quantitation techniques like immunohistochemistry/immunofluorescence, Western blot (WB), and capillary immunoassay, have limitation in accuracy and reproducibility.^23^ Finally, and perhaps most importantly, the loss of specific domains of the protein, crucial for its function, or the preservation of the “phase” of spectrin repeats or other domains surrounding the deletion breakpoint, may have a larger impact than sheer protein quantity,^14,24,25^ and affect the stability and functionality of the protein. Because of the complex relation between dystrophin quantity and phenotype, dystrophin WB data should be evaluated carefully, in conjunction with genotype and the clinical status of the patient, both in clinical practice and clinical research settings. Muscle biopsies with dystrophin assays are especially useful in cases where the clinical picture and genotype are not clearly concordant.

Our findings indicate that only a small proportion of individuals with BMD experienced LoA over their lifetime because only 25% lost ambulation by the age of 60. In this study, we only observed 13 participants aged 60 years or older, leading to uncertainty in LoA risk estimates in this age range. In a recently published, larger, multicentric Italian cohort of n = 943^26^ (which also includes most participants in this study), LoA was slightly earlier: 25% at around 50 years, median (50%) LoA at 69 years. We speculate that the BMD cohort in Padova may be “enriched” by milder cases because of a tendency to perform muscle biopsies with dystrophin quantitation and/or *DMD* MLPA assays in paucisymptomatic males with elevated CK.

We can also reaffirm that the NSAA exhibits a significant decline, even more evident over a prolonged longitudinal follow-up, remaining a reliable outcome measure for BMD trials and long-term efficacy analyses.

Furthermore, our long-term follow-up allowed us to observe a significant decrease in all TFTs and in the 6MWT. The latter holds promise as a tool to objectively measure fatigability by assessing the decrease of distance covered in the last part of the test, although not yet standardized or validated for this purpose.

Another significant practical insight is that BMD individuals with muscle function preserved at, or near normal levels (defined as a NSAA score of 33 or 34 out of 34) and/or those belonging to specific variant groups, mainly del 48 and del x-51, consistently exhibit a “nonprogressive” dystrophinopathy.

When planning a phase IIb-III trial with efficacy as the goal, it might be advisable to exclude these “nonprogressive” individuals, as a drug-induced effect in delaying the progression of muscle weakness may not be apparent. Therefore, the exclusion criteria should be directed toward specific variant groups or, perhaps more appropriately, based on functional criteria at the onset of the study. In fact, we estimated, based on progression rates calculated in various NSAA score categories at baseline (Table 4), that BMD individuals with NSAA scores below 32 at study outset will likely demonstrate muscle weakness progression within a time frame of 1–2 years, which is the typical duration of clinical trials. It may be advisable to evaluate individuals with a “ceiling” 34/34 score at NSAA with more sensitive outcomes (e.g., North Star Assessment for limb girdle Dystrophies, NSAD^27^) to distinguish patients with truly preserved muscle strength from those with subtle weakness.

This study underscores the urgent need to further characterize motor function outcome measures and their applicability in clinical research as effectiveness indicators, especially for interventional trials, in a rapidly changing landscape of clinical research in BMD.

The main limitation of this study was that we did not provide a systematic description of cardiac and respiratory features of the population under study, which we plan to present separately. We did, however, exclude participants with symptomatic cardiomyopathy, who were very few, from functional evaluations described here; and no participants presented with respiratory issues so severe, as to interfere with functional evaluations.

We acknowledge other limitations as well, such as the lack of a fixed and regular number of evaluations, although most assessments were conducted yearly. Some individuals with severe phenotypes and subsequently reduced mobility may have been more easily lost to neurology follow-up, because of difficulties traveling and attending clinic visits. This raises a doubt of a relative under-representation of a severe end of the BMD spectrum with this study design. Finally, WB data were obtained from diagnostic assays, rather than in a research setting.

In conclusion, our data refine genotype-phenotype correlations in BMD over an average observational period of 6 years and enhance our understanding of disease progression. This nuanced insight is crucial for informing the design and interpretation of clinical trials in BMD. Our findings also suggest useful inclusion and exclusion criteria for trials aimed at demonstrating efficacy in motor outcomes. Notably, we quantified the decline in several practical and validated motor outcome measures, providing essential data for power calculations in clinical trials. The extended observational period of the data presented here will also serve as an external comparator for long-term efficacy analyses, especially for real-world data following the approval of new treatments.

## Glossary

6MWT: 6-minute walk test
BMD: Becker muscular dystrophy
CK: creatinkinase
DCM: Dilated cardiomyopathy
DMD: Duchenne muscular dystrophy
IB: immunoblot
LoA: loss of ambulation
NSAA: North Star Ambulatory Assessment
TFT: timed function test
TRISE: time to rise from the floor
TSTEPS: time to climb 4 standard step
TTRW10: timed to walk/run 10 m
